# Glucose- and Bile Acid-Stimulated Secretion of Gut Hormones in the Isolated Perfused Intestine Is Not Impaired in Diet-Induced Obese Mice

**DOI:** 10.3389/fendo.2022.884501

**Published:** 2022-05-04

**Authors:** Jenna E. Hunt, Jens J. Holst, Sara L. Jepsen

**Affiliations:** ^1^ Department of Biomedical Sciences, Faculty of Health and Medical Sciences, University of Copenhagen, Copenhagen, Denmark; ^2^ Novo Nordisk Foundation Center for Basic Metabolic Research, Faculty of Health and Medical Sciences, University of Copenhagen, Copenhagen, Denmark

**Keywords:** diabetes, diet-induced obesity, gut hormones, obesity, incretin, PYY, somatostatin

## Abstract

**Purpose:**

Decreased circulating levels of food-intake-regulating gut hormones have been observed in type 2 diabetes and obesity. However, it is still unknown if this is due to decreased secretion from the gut mucosal cells or due to extra-intestinal processing of hormones.

**Methods:**

We measured intestinal hormone content and assessed morphological differences in the intestinal mucosa by histology and immunohistochemistry. Secretion of hormones and absorption of glucose and bile acids (BA) were assessed in isolated perfused mouse intestine.

**Results:**

GIP (glucose-dependent insulinotropic polypeptide) and SS (somatostatin) contents were higher in the duodenum of control mice (p < 0.001, and <0.01). Duodenal GLP-1 (glucagon-like peptide-1) content (p < 0.01) and distal ileum PYY content were higher in DIO mice (p < 0.05). Villus height in the jejunum, crypt depth, and villus height in the ileum were increased in DIO mice (p < 0.05 and p = 0.001). In the distal ileum of DIO mice, more immunoreactive GLP-1 and PYY cells were observed (p = 0.01 and 0.007). There was no difference in the absorption of glucose and bile acids. Distal secretion of SS tended to be higher in DIO mice (p < 0.058), whereas no difference was observed for the other hormones in response to glucose or bile acids.

**Conclusion:**

Our data suggest that differences regarding production and secretion are unlikely to be responsible for the altered circulating gut hormone levels in obesity, since enteroendocrine morphology and hormone secretion capacity were largely unaffected in DIO mice.

## Introduction

Glucose-dependent insulinotropic polypeptide (GIP), glucagon-like peptide-1 (GLP-1), peptide tyrosine tyrosine (PYY), and somatostatin (SS) are all important regulators of glucose homeostasis and body weight (1–4). GIP and GLP-1 are incretin hormones (5) while PYY, but also GLP-1, reduces appetite and food intake (6–8), and SS is the main paracrine regulator of the secretion of these hormones (4, 9, 10). The decreased secretion of GLP-1 and PYY is associated with the development of type 2 diabetes (T2D) and obesity (1, 11–16), and upregulating their secretion as observed in gastric bypass surgery can lead to remission/improvement of both obesity and T2D (17–20). The notion of impaired secretion in obesity is based on measurements of lower plasma concentrations, and an explanation for this decrease has not been found. Thus, it is unclear whether the gut secretory capacity is altered, or whether the changing levels are due to deficient stimulation and/or extra-intestinal mechanisms such as degradation, metabolism, and differential elimination rates.

In this study, we sought to elucidate whether there are differences in gut hormone storage (tissue concentration and immunoreactive cells), absorption of nutrients, and secretory capacity of gut hormones between diet-induced obese (DIO) mice and age-matched control mice. Specifically, we measured small intestinal contents of GIP, GLP-1, PYY, and SS in the duodenum, jejunum, proximal ileum, and distal ileum and evaluated the height and depth of villi and crypts and the number of immunoreactive GIP, GLP-1, PYY, and SS cells. Using the perfused proximal and distal small intestine, we measured the stimulated absorption of glucose and bile acids (BA) and the secretion of hormones in DIO and control mice in response to glucose (proximal gut) or BA (distal gut).

## Material and Methods

### Animals

Male DIO mice (DIO-B6) and controls (C57BL/6NTac) (27–31 weeks, DIO: 46–59 g, controls: 30–36 g) were purchased from Taconic (Hudson, NY). DIO mice were housed 3–4 mice per cage, whereas the control mice were single housed. They were acclimatized for a month before the experiments and were under a 12-h light/dark cycle with free access to water and chow. The control mice had free access to a 10% kcal fat control diet, and the DIO mice had access to a 60% kcal high-fat diet (cat. no. D12450J and cat. no. D12492 respectively, Research Diets, Inc., New Brunswick, USA). All mouse experiments were conducted in accordance with the recommendations of the National Institutes of Health (publication number 85-23) and the European Convention for the Protection of Vertebrate Animals used for experimental and other scientific purposes (Council of Europe No. 123, Strasbourg 1985) as well as in accordance with the guidelines of the Danish legislation governing animal experimentation (1987). Experiments were carried out with permission from the Danish Animal Experiments Inspectorate (2018-15-0201-01397) and our local Institutional Animal Care and Use Committee (Department of Experimental Medicine, protocol no. P19-220 and P-20-067).

### Protein Extraction

Tissue samples were harvested before and after the perfusion of the distal small intestine. The following tissue samples of 1.5 cm in length were excised after the perfusion: duodenum (the first 1.5 cm after pylorus), jejunum (1.5 cm following the duodenum), and distal ileum (starting 2.5 cm proximally from the caecum). Only the proximal ileum was isolated before the operation since that was the only piece which was removed before the perfusion starts. All samples were stored in RNAlater (cat. no. R0901, Sigma-Aldrich, St. Louis, MA, USA) at -20°C until protein extraction. The tissue samples were weighed and homogenized in 1 ml 1% (v/v) trifluoroacetic acid (TFA) (cat. no. TS-28904, Thermo Fisher Scientific, Waltham, MA, USA) with a 5-mm steel bead (TissueLyser, Qiagen Instruments AG, Hombrechtikon, Switzerland) at 30 Hz two times for 4 min. Next, samples were left to stand for 1 h at room temperature and cleared by centrifugation (6,000 rpm, 10 min). A volume of 25 µl of the supernatant was used for Pierce BCA Protein Assay for determination of the total protein concentration according to the manufacturers’ manual (cat. no. 23227, Thermo Scientific, USA). The rest of the supernatant was purified using Sep-Pak pH-resistant tc18 cartridges (cat. no. WAT036810, Waters, Milford, MA, USA), and peptides were eluted in 70% ethanol + 0.1% TFA. The eluate was dried under a gentle stream of compressed air overnight. Samples were reconstituted in 1 ml of assay buffer containing 80 mM phosphate buffer, 0.1% human serum albumin, and 10 mM EDTA, pH 7.5, and left to stand for 30 min before analysis by radioimmunoassay (RIA).

### Histology and Immunohistochemistry

Formalin-fixed tissue from DIO (n = 5–6) and control mice (n = 5) was dehydrated and paraffin-embedded. The tissue was cut (4 μm) and stained with hematoxylin/eosin. The average villus height and crypt depth were approximated by measuring these parameters in at least 20 well-oriented villi and crypts per section. All measurements were made from histological photographs taken with a light microscope connected to a camera (Zeiss Axio Lab.A1, Brock & Michelsen, Birkeroed, Denmark) and Zeiss ZEN lite software (Carl Zeiss Microscopy GmbH, Göttingen, Germany). Immunohistochemistry (IHC) was carried out using the primary antibodies GIP (95235, in-house, diluted 1:6,000), GLP-1 (2135, in-house, 1:10,000), PYY (EUD5201, Acris Antibodies, Herford, Germany, 1:40,000), and SS (1759, in-house, 1:15,000). For antigen retrieval, the sections were boiled in a microwave for 15 min in EDTA-buffer pH 9, followed by a pre-incubation in 2% bovine serum albumin (BSA) for 10 min. The GIP, GLP-1, and SS antibodies were diluted in 2% BSA and incubated for 1 h at room temperature whereas PYY was incubated overnight at 4°C. To amplify the reactions, the sections were incubated for 40 min with biotinylated secondary antibody immunoglobins: GIP, GLP-1, and SS with Goat anti-Rabbit (BA-1000, Vector Laboratories, Burlingame, CA, USA, 1:200) and PYY with Goat anti-guinea pig (BA-7000, Vector Laboratories, Burlingame, CA, USA, 1:200). Afterward, endogenous peroxidase was blocked with 3% hydrogen peroxidase. A third layer was formed by a preformed avidin and biotinylated horseradish peroxidase macromolecular complex: for GIP, GLP-1, and SS (Elite ABC, PK-6100, Vector Laboratories) for 30 min and for PYY (ABC, PK-4000, Vector Laboratories). The reaction was developed by the use of 3,3-diaminobenzidine (DAB+) (SK-4105 and DAB SK-4100 for PYY, Vector Laboratories) for 15 min and counterstained with Mayer’s hematoxylin. Immunopositive cells were counted per whole intestinal section using a light microscope, and the observer was blinded as to the origin of the section.

### Intestinal Perfusions

Mice were anesthetized with intraperitoneal injection of Ketamine/Xylazine (0.1 ml/20 g) (Ketamine 90 mg/kg (Ketaminol Vet.; MSD Animal Health, Madison, USA) and Xylazine 10 mg/kg (Rompun Vet.; Bayer Animal Health, Germany). The colon, stomach, and spleen were tied off and removed, and the kidneys were ligated. A tube was placed in the proximal opening of the intestine, allowing luminal perfusion of 37°C isotonic saline (0.9% NaCl), at a rate of 0.035 ml/min. Hereafter, a catheter (BD Insyte Autoguard, 24 GA 0.75 IN, 0.7 × 19 mm, BD, Denmark) was placed in the abdominal part of the aorta and in the portal vein to collect venous effluents every minute using a fraction collector. The mice were euthanized by perforation of the diaphragm. The intestine was perfused at a flow rate of 2.5 ml/min using a modified Krebs-Ringer bicarbonate buffer containing 0.1% BSA (Merck KGaA), 5% Dextran T-70 (Dextran Products Limited, Toronto, Canada), 3.5 mmol/l glucose, and 5 mmol/l each of pyruvate, fumarate, and glutamate as well as 10 μmol/l 3-isobutyl-1-methylxanthine (IBMX) (cat. no. 5879, Sigma-Aldrich, USA) and 5 mmol/l Vamin (a mixture of essential and non-essential amino acids; Fresenius Kabi, Copenhagen, Denmark). The buffer was pH adjusted to ~7.5, heated to 37°C, and gassed with a 95% O_2_/5% CO_2_ mixture. The experiments were started after an equilibrium period of 25 min.

The proximal small intestine was luminally stimulated by instillation of ~20% glucose dissolved in saline at a flow rate of 0.135 ml/min for the first 3 min and hereafter 0.035 ml/min until 15 min had passed. The distal small intestine was likewise stimulated through the lumen but with the BA mixture with a final luminal concentration of 9 mM BA, containing 1 mM of each sodium cholate (cat. no. C1254), sodium glycocholic acid (cat. no. G7132), sodium taurocholate (cat.no. 86339), sodium deoxycholate (cat.no. D6750), sodium glycodeoxycholate (cat. no. G9910), sodium taurodeoxycholate (cat. no. T0875), sodium chenodeoxycholate (cat. no. C8261), sodium glycochenodeoxycholate (cat. no G0759), and sodium taurochenodeoxycholate (cat. no. T6260) all purchased from Sigma-Aldrich, USA.

### Glucose and Bile Acid Absorption From the Perfused Mouse Intestine

Glucose and BA absorption was measured from the venous effluent from perfusions in samples for every minute. Glucose was measured with a handheld glucometer (Accu-Chek Mobile, cat. no. 05874149001, Roche Diagnostics, Mannheim, Germany). Total BA was measured using an enzymatic assay according to the manufacturers’ protocol (cat. no. STA-631, Cell Biolabs Inc., USA).

### Biochemical Measurements

Tissue extracts and perfusion effluent were measured by radioimmunoassay (RIA). Total GIP(1-42) was measured with an in-house-developed RIA using synthetic mouse GIP as standard (cat. no. 027-27, Phoenix Pharmaceuticals, Burlingame, CA, USA), a side-viewing antiserum directed against the N-terminal part of the peptide (code. no. 98170-4), and ^125^I-labeled human GIP(1-42) tracer (cat. no. Nex402, PerkinElmer, Waltham, MA, USA). Total GLP-1 (7-36amide) was measured with an in-house-developed RIA, based on a C-terminally directed antiserum specific for the amidated GLP-1 form (code no. 89390) (21). The standard was synthetic GLP-1 7–36NH_2_ (cat. no. H-6795-GMP, 4081700, Bachem, Frechen, Germany), and the tracer was monoiodinated ^125^I-labeled GLP-1 (7–36NH_2_) (a gift from Novo Nordisk A/S, Bagsværd, Denmark). SS was measured using an in-house-developed RIA (code no. 1758) (22), which uses SS-14 as standard (cat. no. H-1490, Bachem, Germany), a rabbit antiserum raised against synthetic cyclic SS, recognizing both isoforms of SS (SS-14 and SS-28), and a ^125^I-labeled SS tracer (cat. no. NEX389010UC, PerkinElmer, Denmark). Total PYY was measured with a porcine antiserum (cat. no T-4093, Bachem, Germany); it should be noted that porcine and murine PYY share the same amino acid sequence (23). The antibody used recognizes PYY_1/3–36_ and PYY_1/3–34_ equally (24). The standard was synthetic rat/mouse/porcine PYY (cat. no. H-6045, Bachem, Germany), and the tracer was ^125^I-labeled porcine PYY for the measurement of perfusion samples and ^125^I-labeled human PYY for the tissue extracts (cat no. NEX2400 and NEX3410, respectively, PerkinElmer, Denmark). Free and bound peptides were separated with plasma-coated charcoal (cat. no. 1.02186.0250, Merck, Germany). Antibody/antigen-bound radioactivity was counted by a gamma counter (Wizard^2^, Automatic Gamma Counter, PerkinElmer, Denmark), and hormone levels were calculated by a four-parameter interpolation to the different standard curves.

### Data Presentation and Statistical Analysis

All data are presented as mean ± SEM, and differences resulting in a p value < 0.05 were considered significant. The graphs and statistical analysis were made using GraphPad Prism 8 (GraphPad, La Jolla, USA). Differences in total protein content were evaluated using a two-tailed unpaired Student’s t-test comparing two independent groups. Histology and IHC estimates were compared using 2-way ANOVA followed by Bonferroni’s multiple-comparison test. Glucose and BA output after stimulation was calculated by subtracting the baseline levels from the output during stimulation until the end of experiment. The baseline subtracted output was multiplied with the flow (2.5 ml/min) to obtain µmol/min. The output (secretion) of hormones in the venous effluents are presented as fmol/min calculated by multiplying the perfusion flow (2.5 ml/min) with the hormone concentration in pmol/L. Differences in the hormone output were based on average baseline levels (first 1–10 min of experiment), and the average baseline subtracted the output during stimulation (11–40 min for the proximal perfusions and 11–50 min for the distal perfusions). Differences in secretion at baseline and during stimulation, as well as differences in the length of the perfused segments, were evaluated by two-tailed unpaired Student’s t-tests. To test differences in the responses normalized to the length of the intestine, the average hormone output (fmol/min) from the start of stimulation to the end of that experiment was divided by the length of the intestine from each individual experiment and evaluated by a two-tailed unpaired Student’s t-test.

## Results

### Hormone Content in Intestinal Segments

Hormone content was normalized to tissue weight (25, 26) as well as to the total protein measured by BCA.

**Figure 1:**
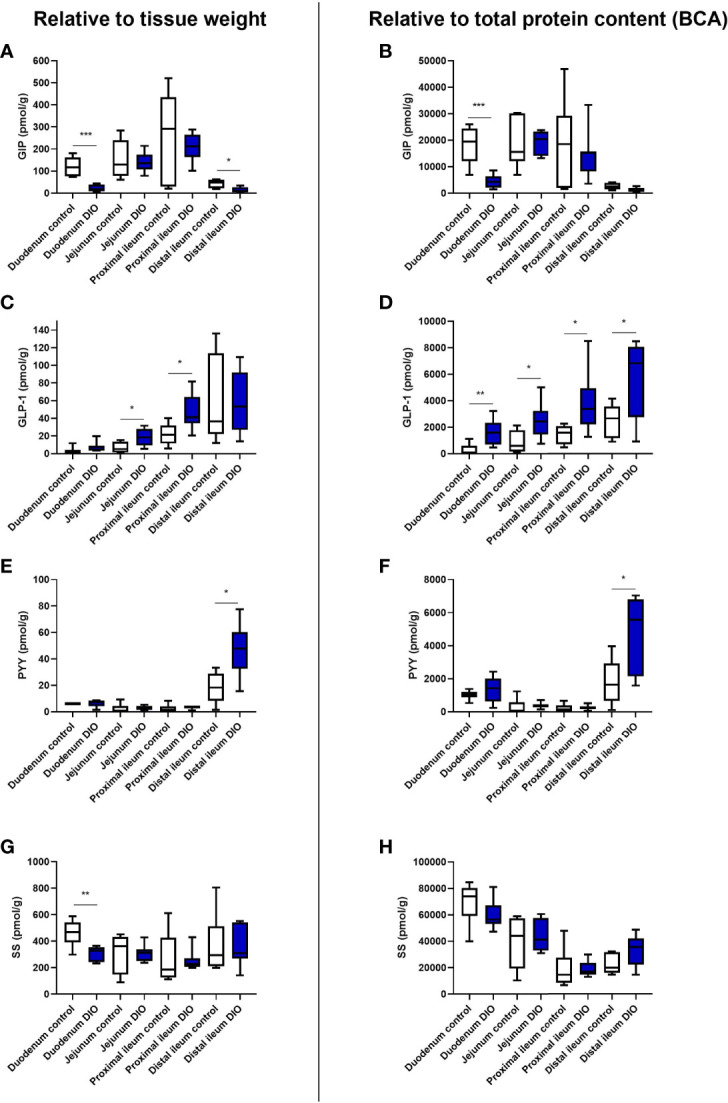
Protein content of GIP, GLP-1, SS, and PYY in the small intestine from control mice and DIO mice. Hormone concentrations of total GIP **(A, B)**, total GLP-1 **(C, D)**, total PYY **(E, F)**, or total SS **(G, H)** were measured in different segments of the small intestine from tissue isolated from either control mice (white bars, n = 6) or DIO mice (blue bars, n = 7). The hormone concentration was either normalized to the wet weight of the tissue **(A, C, E, G)** or to total protein content **(B, D, F, H)**. Data are presented as mean ± SEM, and statistical significance was evaluated by a two-sided, unpaired Student’s t-test, *p < 0.05, **p < 0.01, ***p < 0.001.

GIP levels were markedly lower in DIO mice in the duodenum whether normalized to tissue weight or protein content (p = 0.003, **Figures 1A, B**), but levels were similar in the jejunum and the proximal ileum (per weight p = 0.8 and p = 0.5, respectively, **Figure 1A**, and per protein p = 0.9 and 0.6, respectively, **Figure 1B**). DIO levels were again lower in the distal ileum (per weight, p = 0.019, **Figure 1A**, and per protein p = 0.065, **Figure 1B**).

GLP-1 levels were higher in the jejunum and proximal ileum of DIO mice (jejunum per weight p = 0.02; proximal ileum p = 0.017, **Figure 1C**). A trend to higher GLP-1 levels was observed in the duodenum (p = 0.07), and no significant difference was observed in the distal ileum (p = 0.9). When normalizing to total protein content, the levels of GLP-1 were significantly higher throughout the small intestine in DIO mice compared to control mice (duodenum; p = 0.009, jejunum; p = 0.03, proximal ileum; p = 0.03, and distal ileum; p = 0.03, **Figure 1D**).

PYY levels were low throughout the proximal small intestine in both control and DIO mice (per weight <6 pmol/g, **Figure 1E**). In the distal ileum, there was more PYY in DIO mice (p = 0.015, **Figures 1E, H**), while in the duodenum, jejunum, and proximal ileum the levels were similar in control and DIO mice (per protein, p = 0.3, p = 0.6, and p = 0.7, respectively, **Figure 1F**).

For somatostatin, duodenal levels were lower in DIO mice (per wet weight, p = 0.005, **Figure 1G**), whereas no difference was observed for the jejunum (p = 0.9), proximal ileum (p = 0.9), or distal ileum (p = 0.8). Normalizing to protein, there were no differences between the groups in the duodenum (p = 0.2), jejunum (p = 0.7), proximal ileum (p = 0.9), or distal ileum (p = 0.07, **Figure 1H**).

### Impact of DIO on Jejunal and Ileal Villus and Crypt Morphology

Villus height was increased in DIO mice compared to control mice in the jejunum (control 450.9 ± 15.9 µm vs. DIO 553.8 ± 16.2 µm, p = 0.03, data not shown) and proximal ileum (control 479.5 ± 20.9 µm vs. DIO 563.6 ± 35.9 µm, p = 0.02 µm), as was the crypt depth both in the proximal ileum (control 76.5 ± 2.9 µm vs. DIO 87.6 ± 6.4 µm, p = 0.008) and in the distal ileum (64.25 ± 1.8 µm vs. DIO 80.41 ± 3.55 µm, p = 0.02). No difference was seen in jejunal crypt depth (p = 0.22).

**Figure 2:**
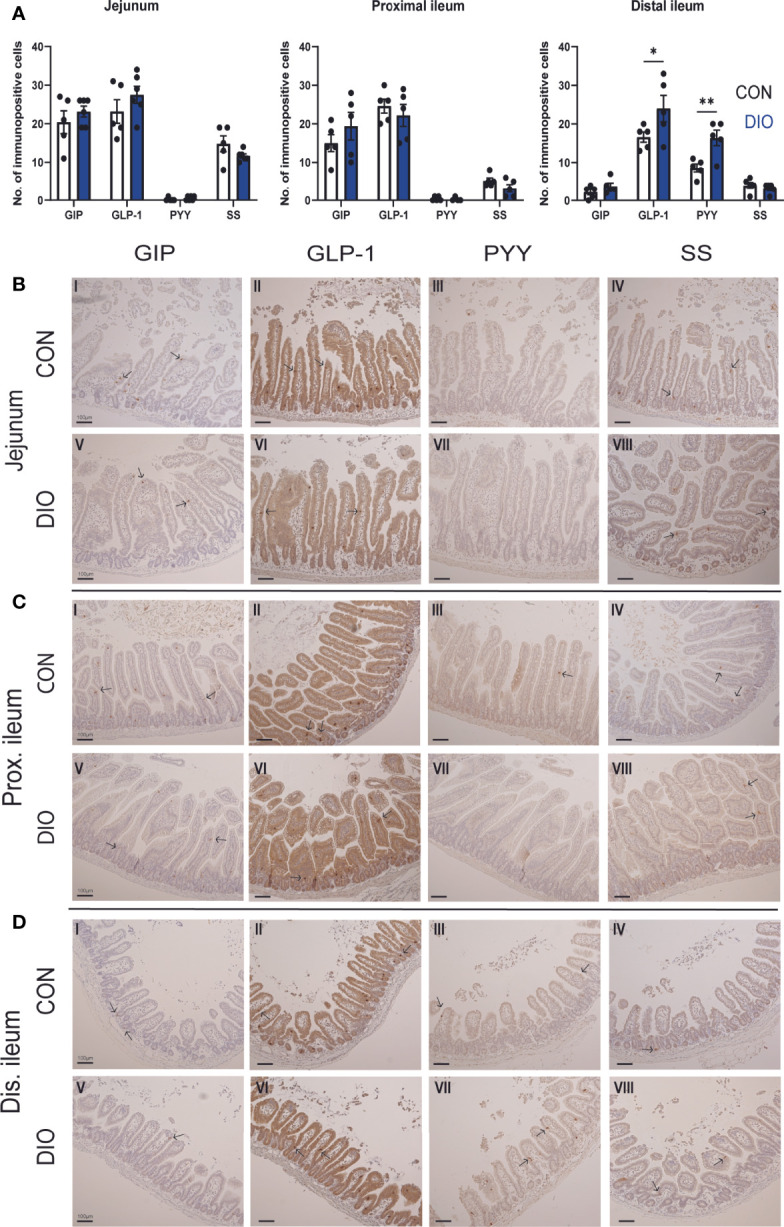
Immunohistochemical comparison of K-, L- and D-cells in the jejunum and ileum of control vs. DIO mice. **(A)** The number of GIP, GLP-1, PYY, and SS immunopositive cells in the jejunum and proximal and distal ileum in control mice (white bars, n = 5) or DIO mice (blue bars, n = 5–6). Data are presented as mean ± SEM, and statistical significance was evaluated by 2-way ANOVA followed by Bonferroni’s multiple-comparison test, *p < 0.05 and **p < 0.01 **(B–D)** Histological appearances and IHC for GIP, GLP-1, PYY, and SS in the jejunum, proximal and distal ileum of control vs. DIO mice. Scale bar corresponds to 100 µm.

Gut hormone cell types were investigated using IHC staining for GIP, GLP-1, PYY, and SS. In both cases, GLP-1 immunopositivity was stably present in the jejunum and ileum (**Figure 2A**). GIP and SS immunopositivity decreased distally, while PYY immunopositivity increased distally. In the jejunum and proximal ileum, there were no differences in GIP, GLP-1, PYY, or SS immunopositivity between control and DIO mice. In the distal ileum, GLP-1 and PYY immunopositivity was increased (p = 0.01 and 0.007, **Figure 2A**), but GIP and SS remained unchanged. Representative images of the stainings are shown in **Figures 2B–D**.

### Absorption of Glucose and Glucose-Induced GIP, GLP-1, and SS Secretion From Isolated Perfused Proximal Small Intestine

**Figure 3:**
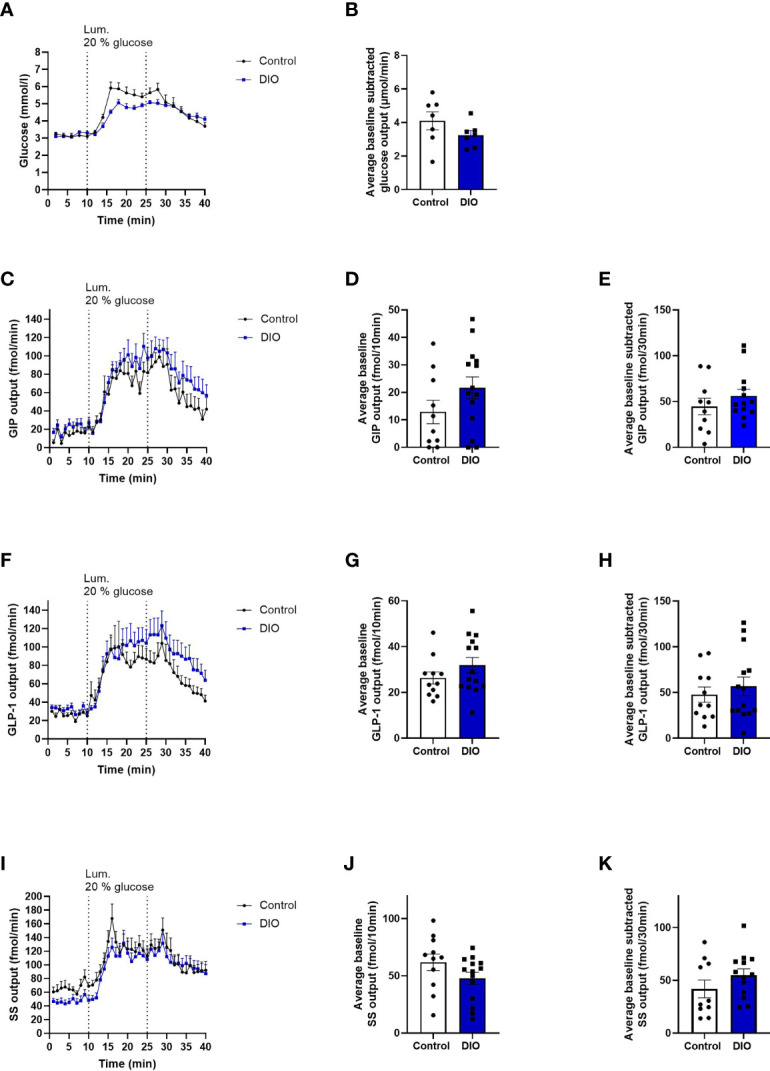
Absorption of glucose and secretion of GIP, GLP-1, and SS are the same in isolated perfused proximal small intestines from control and DIO mice. **(A)** Glucose concentration (mmol/L) measured in the venous effluents from control mice (black line, n = 7) or DIO mice (blue line, n = 7). **(B)** Average baseline subtracted glucose output (absorption) from 11 to 40 min in control mice (white box) and DIO mice (blue box) **(C, F, I)** GIP, GLP-1, and SS output in the venous effluent in control mice (black line, n = 10–11) and DIO mice (blue line, n = 14) in response to 20% luminal glucose infusion. **(D, G, J)** Average baseline GIP, GLP-1, and SS output from 1 to 10 min in control mice (white box) and DIO mice (blue box). **(E, H, K)** Average baseline-subtracted GIP, GLP-1, and SS output during 20% luminal glucose infusion from 11 to 40 min in control mice (white box) and DIO mice (blue box). Each dot represents outputs from each experiment. Data are presented as mean ± SEM, and statistical significance was evaluated by a two-sided, unpaired Student’s t-test.

The length of the perfused segment was 12.7% longer in DIO mice (controls: 11.36 ± 0.4 cm n = 11. DIO mice: 12.71 ± 0.4 cm, n = 14, p = 0.0054). Glucose absorption was similar in the two groups. (Baseline-subtracted venous output during and after glucose infusion_11-40min_: control mice 4.1 ± 0.5 µmol/min, DIO mice: 3.25 ± 0.3 µmol/min, p = 0.1, **Figures 3A, B**). Normalized to the length of the intestine, absorption was insignificantly higher in control mice (controls: 0.37 ± 0.05 mmol/cm*min, DIO mice: 0.26 ± 0.02 µmol/cm*min, p = 0.058, n = 7).

GIP output (effluent concentration × perfusion flow) did not differ between control mice and DIO mice during baseline_1-10 min_ (p = 0.15, **Figures 3C, D**) or in response to luminal glucose infusion (control output_11-40 min_: 44.6 ± 9.1 fmol/30 min, DIO mice: 56.2 ± 7.3 fmol/30 min, p = 0.3, **Figures 3C, E**) or on normalization to intestinal length (average GIP output_11-40 min_/cm: 3.96 ± 0.8 fmol/cm *min, DIO mice: 4.5 ± 0.6 fmol/cm*min, p = 0.6, n = 11–14, data not shown).

GLP-1 output did not differ at baseline or after glucose infusion between control mice and DIO mice (control mice baseline_1-10 min_: 26.3 ± 2.7 fmol/10 min, DIO mice baseline_1-10 min_: 31.9 ± 3.5 fmol/10 min, p = 0.2, **Figures 3F, G**; after glucose in control mice_11-40 min_: 47.7 ± 8.3 fmol/30 min, DIO mice: 56.76 ± 10.1 fmol/30 min, p = 0.5, **Figures 3F, H**). The same was true after normalization to intestinal length (GLP-1 output_11-40 min_, control mice: 4.3 ± 0.8 fmol/cm*min. DIO mice: 4.4 ± 0.7 fmol/cm*min, p = 0.9, n = 11–14, data not shown).

SS output did not differ between the two groups at baseline or after glucose infusion (control baseline_1-10 min_: 61.8 ± 7.3 fmol/10 min, DIO mice: 47.8 ± 5.3 fmol/10 min, p = 0.1, **Figures 3I, J**. Control SS output after glucose infusion_11-40 min_: 41.9 ± 8.3 fmol/30 min, DIO mice: 54.9 ± 5.9 fmol/30 min, p = 0.8, **Figures 3I, K**). Expressed per cm intestinal length, there was still no difference in glucose-induced SS output (SS output_11-40 min_ normalized to length, control mice: 3.6 ± 0.7 fmol/cm*min. DIO mice: 3.9 ± 0.6 fmol/cm*min, p = 0.7, n = 11–14, data not shown).

### BA-Induced Secretion From Perfused Distal Small Intestine in DIO and Control Mice

A BA mixture, composed of nine different conjugated and unconjugated bile acids, was chosen instead of glucose, since BAs have been shown to be potent stimulators of GLP-1 and PYY secretion in the most distal part of the intestine (27–29). while glucose mainly is absorbed in the proximal small intestine (30, 31).

The lengths of the perfused segments were similar in the two groups (control mice: 12.4 ± 0.4 cm, n = 5. DIO mice: 13.3 ± 0.8 cm, n = 6, p = 0.3, data not shown).

**Figure 4:**
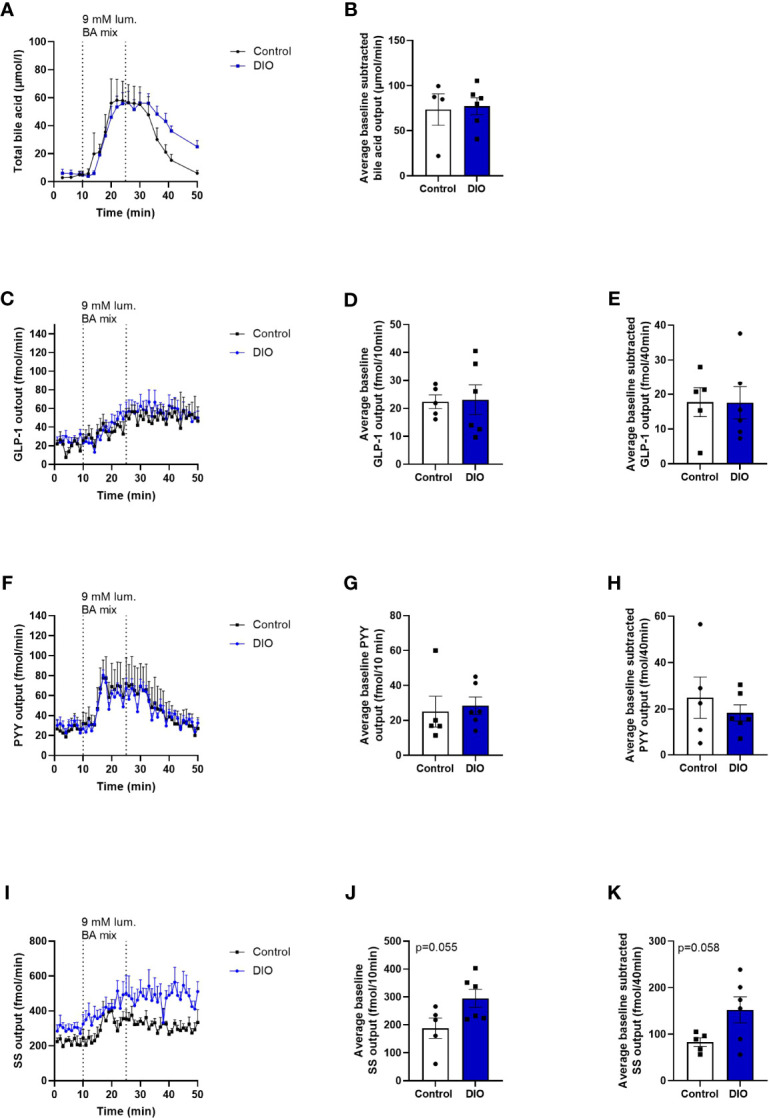
Absorption of BA and the secretion of GLP-1 and PYY are similar, but SS secretion tends to be higher in DIO mice in isolated perfused distal small intestine. **(A)** BA concentration (mmol/L) measured in the venous effluents from control mice (black line, n = 4) or DIO mice (blue line, n = 6). **(B)** Average baseline-subtracted BA concentration (absorption) in the venous effluent from 11 to 50 min in control mice (white box) and DIO mice (blue box). **(C, F, I)** GLP-1, PYY, and SS output in the venous effluent in control mice (black line, n = 5) and DIO mice (blue line, n = 6) in response to luminal BA mix infusion. **(D, G, J)** Average baseline GLP-1, PYY, and SS output from 1 to 10 min in control mice (white box) and DIO mice (blue box). **(E, H, K)** Average baseline-subtracted GLP-1, PYY, and SS output during luminal BA infusion from 11 to 50 min in control mice (white box) and DIO mice (blue box). Each dot represents outputs from each experiment. Data are presented as mean ± SEM, and statistical significance was evaluated by a two-sided, unpaired Student’s t-test.

BA absorption was similar in DIO and control mice (BA concentrations in the venous effluent × perfusion flow; control mice: 73.5 ± 17.4 µmol/min, DIO mice: 77.2 ± 9.3 µmol/min, p = 0.8, **Figures 4A, B**).

Baseline GLP-1 output did not differ between control and DIO mice in the distal intestine (control mice baseline output_1-10min_: 22.4 ± 2.4 fmol/10 min, DIO mice: 23.1 ± 5.3 fmol/10 min, n = 5–6, p = 0.7, **Figures 4C, D**), and neither did the baseline subtracted output after BA infusion (control mice output_11-50min_: 24.9 ± 8.9 fmol/40min, DIO mice: 18.4 ± 3.5 fmol/40min, p = 0.9, **Figure 4E**).

Likewise, PYY output did not vary between the two groups at baseline or after luminal infusion of the BA mix (average baseline PYY output_1-10min_ in control mice: 25 ± 8.9 fmol/10 min, DIO mice: 28.4 ± 4.9 fmol/10 min, n = 5–6, p = 0.7, **Figures 4F, G**, PYY output after BA mix_11-50min_ control mice: 24.9 ± 8.9 fmol/40 min, DIO mice: 18.4 ± 3.5 fmol/40 min, p = 0.5, **Figure 4H**).

Somatostatin output tended to be higher at baseline and after BA infusion in DIO mice compared to control mice (baseline SS output_1-10min_ in control mice: 187.9 ± 36.3 fmol/10 min, DIO mice: 295 ± 32.3 fmol/10 min, n = 5–6, p = 0.055, **Figures 4I, J**, SS output after BA mix_11-50min_: 82.9 ± 9 fmol/40 min, DIO mice: 152.5 ± 28 fmol/40 min, p = 0.058, **Figure 4K**). However, the fold increase from baseline (1–10 min) to the average output from stimulation to the end of experiment (11–50 min) was the same in the two groups (1.533 ± 0.09, DIO mice: 1.527 ± 0.11, p = 0.96, n = 5–6).

## Discussion

Alterations in gut hormone secretion have been associated with the pathogenesis of obesity, and attenuated responses of GIP, GLP-1, and PYY have been reported both in humans and in rodent models (32–34). A model often used to study the pathogenesis of obesity is the DIO mice in which obesity and eventually T2D are induced by a diet enriched with caloric content, also known as a Western diet. The model gradually tends to worsen their phenotype, making it an appropriate model for the study of the impact of different stages of obesity, and at the same time it mimics the development seen in humans. However, it is also clear that the age of the mice should be chosen with care depending on the stage of obesity/T2D selected for the study. In this study, mice close to the weight gain maximum were used and the mice may thus be closer to a pre-diabetic state. The relative contributions of T2D or obesity per se regarding disturbances in the secretion of the glucose-regulating hormones are a subject of ongoing discussion. However, with respect to GLP-1, it was convincingly shown that the incretin effect is gradually depressed with increasing degree of overweight, rather than being associated with the development of T2D (35), and therefore mice close to their maximum weight gain were chosen for this study. Evaluating the local secretory mechanisms of gut hormones in human studies is difficult, and elucidation of this has to rely on experimental models such as immortalized cell lines, primary isolated mucosal cultures, or organoids [reviewed regarding L-cells in (36)]. These models do not reliably reflect *in vivo* conditions, and it is impossible to relate the magnitude of the secretory responses to the *in vivo* situation. In the isolated perfused intestine, as used here, the gut is kept intact, thus maintaining the polarization of the cells and their normal vascular supply as well as neuronal and paracrine communications. The model is ideal for investigations of the response to intra-luminal stimulations, as they would occur *in vivo*, but without the problems of *in vivo* experiments, such as high degradation rate of hormones in the circulation and limited amount of plasma available in rodent *in vivo* settings. Furthermore, due to the fact that the samples are collected before entering the liver, the model allows accurate measurements of total hormone secretion from the intestinal mucosa as well as absorption from the gut (4, 37–39).

Using this model, our study shows that there is no difference in the basal nor postprandial levels of GIP, GLP-1, or PYY in response to glucose (for GIP and GLP-1) or BA (for GLP-1 and PYY) between control and obese mice. Only SS secretion was increased in the distal small intestine both at baseline and after BA stimulation in DIO mice, but the fold change was the same in DIO and control mice (~1.5-fold increase) in response to a BA mix, indicating a similar responsiveness. The shift in baseline levels of SS in DIO mice could be due to an adaptation to a high-fat diet.

A factor that could affect the secretion of hormones is the absorptive surface of the gut (40), and indeed, we noted that the villus height of the jejunum was shorter in control mice in agreement with a previous study (41). Nevertheless, glucose absorption, which drives at least GLP-1 secretion (40), was the same in the two groups, as was the absorption of BA. Another factor that could affect the secretion of hormone is the number of hormone-secreting cells in the tissue as well as their hormone content. We found an increased number of immunoreactive cells, as well as an increased content of GLP-1 and PYY in some parts of the intestine of DIO mice. However, this did not lead to an increased secretion of the hormones. Interestingly, a study by le Roux et al. showed that DIO mice had lower circulating levels of PYY, in spite of increased tissue contents compared to control mice (42), similar to the findings in the present study. Le Roux et al. proposed that the plasma PYY deficiency might result from impaired PYY release rather than deficient synthesis, although they could not rule out that DIO mice might have an enhanced clearance of PYY. The discrepancy between hormone content and the amount of secretion may also be due to the possibility that not all vesicles secrete their content in response to a stimulus as seen for insulin-containing vesicles (43).

In general, the majority of the studies reporting the altered secretion of gut hormones in diabetes and obesity rely on measurements of circulating gut hormones in plasma, which do not necessarily reflect the actual secretion or the secretory capacity of the endocrine tissue and the intestinal mucosa. Thus, the alteration in plasma concentrations could be a result of changes in stimulus intensities and the absorption surface/rate of nutrients, which was investigated in the present study, as well as to other extra-intestinal events. Based on this study, the altered levels of circulating gut hormones, found in obesity and diabetes, are not due to the intrinsic ability of the gut to respond with adequate hormone secretion to a stimulation. Changes in circulating levels observed *in vivo* are therefore likely to be due to extra-intestinal mechanisms rather than secretory defects of the endocrine organ, which have yet to be interpreted.

## Data Availability Statement

The raw data supporting the conclusions of this article will be made available by the authors, without undue reservation.

## Ethics Statement

The animal study was reviewed and approved by the Danish Animal Experiments Inspectorate (2018-15-0201-01397) and our local Institutional Animal Care and Use Committee (Department of Experimental Medicine, protocol no. P19-220 and P-20-067).

## Author Contributions

SJ and JHo designed the study. SJ and JHu carried out the experiments and interpreted the data. SJ drafted manuscript. JHu and JHo edited and revised the manuscript. All authors contributed to the article and approved the submitted version.

## Funding

SJ was supported by a postdoctoral scholarship from the Danish Diabetes Academy and funded by the Novo Nordisk Foundation, grant number NNF17SA0031406, and by the Independent Research Fund Denmark (ref. no. 0129-00002B). JHu was supported by Aase og Ejnar Danielsens Fond (grant no. 114383). JHo was supported by an unrestricted grant from the Novo Nordisk Center for Basic Metabolic Research (Novo Nordisk Foundation, Denmark grant no. NNF18CC0034900) and another Novo Nordisk grant to JJH (no. NNF15OC0016574) as well as an additional grant from the European Research Council under the European Union’s Horizon 2020 research and innovation program (grant agreement no. 695069-BYPASSWITHOUTSURGERY).

## Conflict of Interest

The authors declare that the research was conducted in the absence of any commercial or financial relationships that could be construed as a potential conflict of interest.

## Publisher’s Note

All claims expressed in this article are solely those of the authors and do not necessarily represent those of their affiliated organizations, or those of the publisher, the editors and the reviewers. Any product that may be evaluated in this article, or claim that may be made by its manufacturer, is not guaranteed or endorsed by the publisher.
